# Supplementary material for: Paired oral clinical specimens reveal the underlying ecology supporting the emergence of inflammophilic microbiome communities
Source: bioRxiv. 2026 Feb 21:2026.02.20.706901. Preprint. [Version 1] doi: 10.64898/2026.02.20.706901 (PMC12934589; doi:10.64898/2026.02.20.706901)
Supplement: Supplement 2 — Figure S1. Species-level ecological composition of plaque and abscess communities. (A) Principal coordinate analysis (PCoA) of species relative abundance values using Bray–Curtis distance with silhouette scores shown alongside the PCoA plot. (B) Hierarchical clustering (Pearson correlation) of log-transformed relative abundances of species separates plaque and abscess communities. Figure S2. Trends in high and medium confidence differentially abundant genera. Persample dot plots of the relative abundance values for selected genera identified as (A) high confidence and (B) medium confidence from Figure 4 show stable trends across the entire cohort. Genera identified as being enriched in the abscess are labeled in red, while those enriched in the plaque are labeled in blue. Figure S3. Fusobacterium abundance measured at different taxonomic levels. Relative abundances of fusobacteria are shown at the (A) phylum, (B) genus, and species levels for (C) Fusobacterium nucleatum and (D) Fusobacterium periodonticum. Total sum scaling (TSS) values are displayed on the left of each panel, with centered log-ratio (CLR) transformed values shown on the right. Figure S4. Comparison of predictive accuracy for genus- and species-level sequence data. In contrast to the random forest model built from genus-level data (Fig. 6), sequence data agglomerated at the species-level was less useful for classifying community type. (A) Receiver operating characteristic (ROC) curves from 4-fold cross-validation exhibit strong performance (area under the curve, or AUC values of 0.56–0.85). (C) Heatmap of feature importance rankings across folds, with top predictors in purple and lower-ranked features in green. The rank of feature importance is shown inside each cell. Figure S5. Algorithmic validation metrics used to select the optimum number of topics for Latent Dirichlet Allocation (LDA) topic-modeling. Four different metrics were used to validate the performance of an LDA model used for (A) speci [file NIHPP2026.02.20.706901v1-supplement-2.pdf]

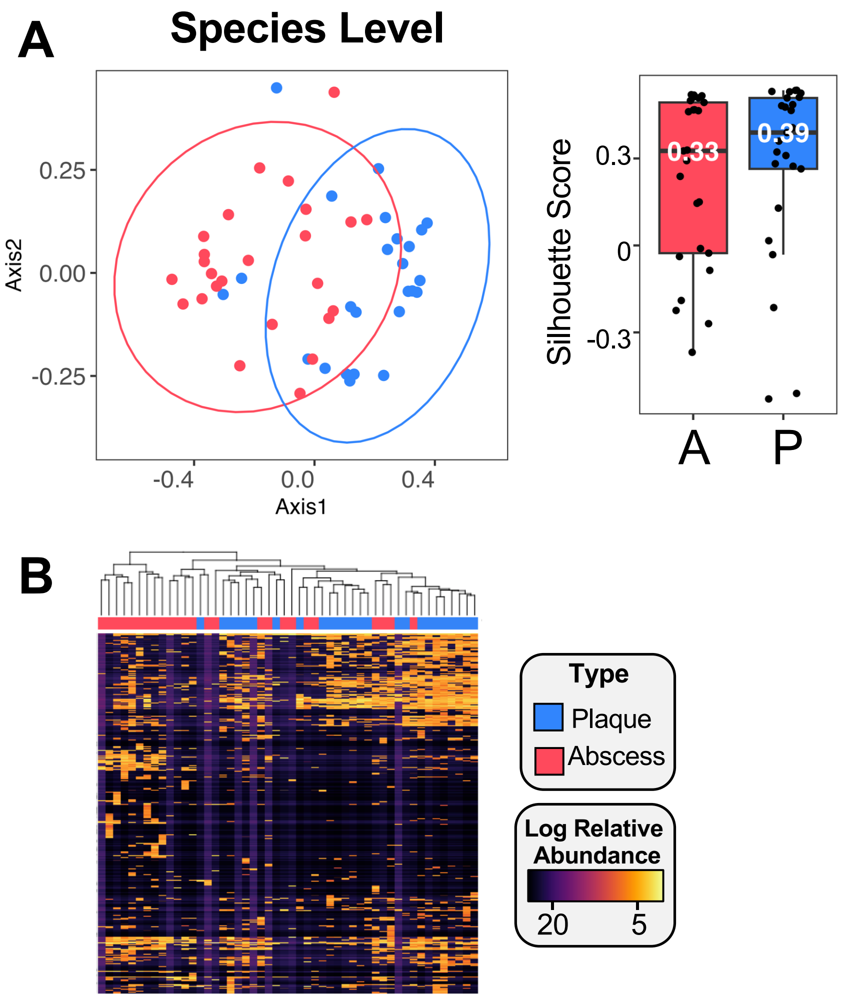
A
Species Level
Axis2
0.25
0.00
-0.25
-0.4
0.0
0.4
Axis1
Silhouette Score
0.3
0
-0.3
0.33
0.39
A
P
B
Type
Plaque
Abscess
Log Relative Abundance
20
5

# Figure S1

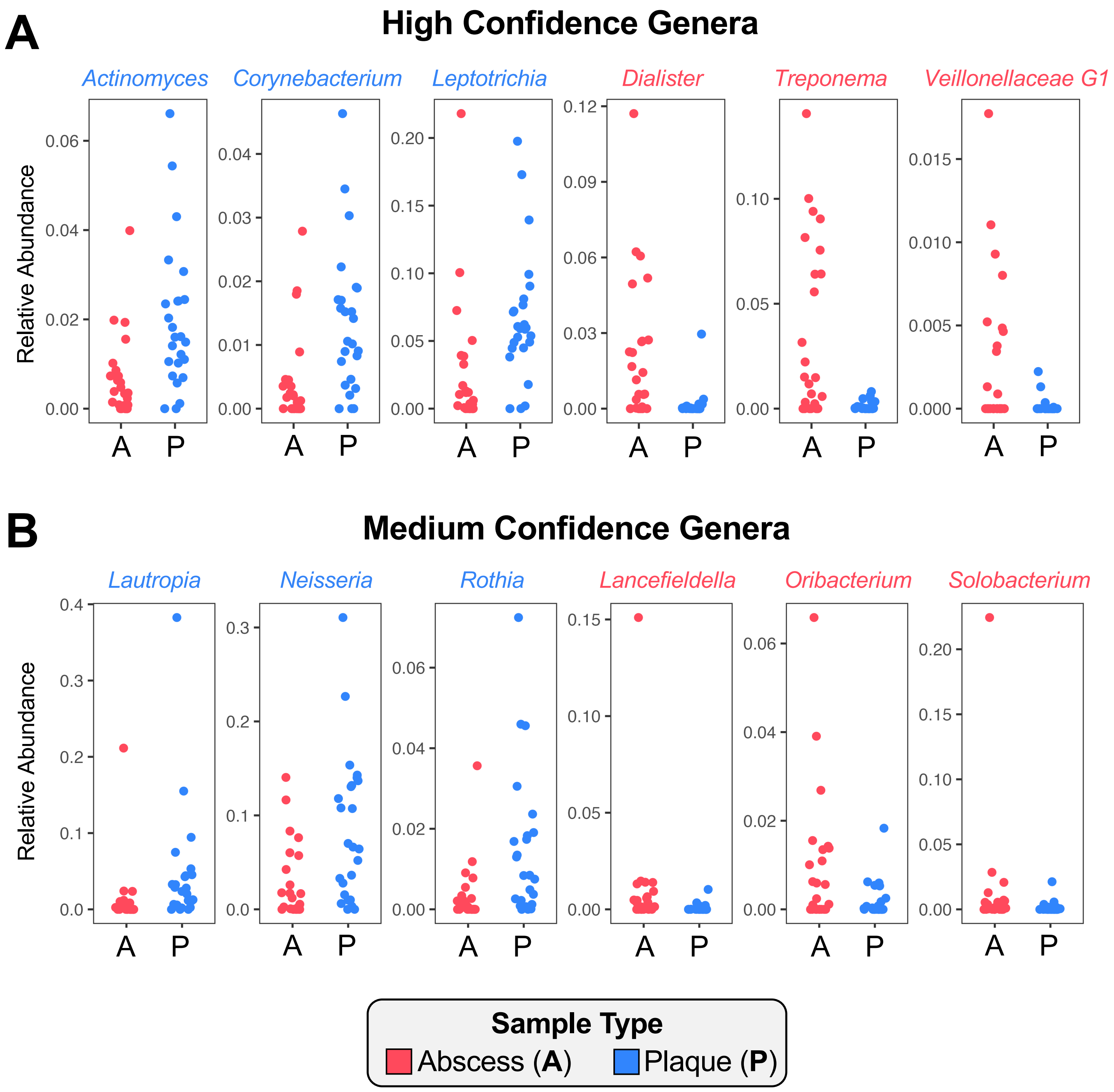
A
High Confidence Genera
Actinomyces
Corynebacterium
Leptotrichia
Dialister
Treponema
Veillonellaceae G1
Relative Abundance
A
P
B
Medium Confidence Genera
Lautropia
Neisseria
Rothia
Lancefieldella
Oribacterium
Solobacterium
Relative Abundance
A
P
Sample Type
Abscess (A)
Plaque (P)

**Figure S2**

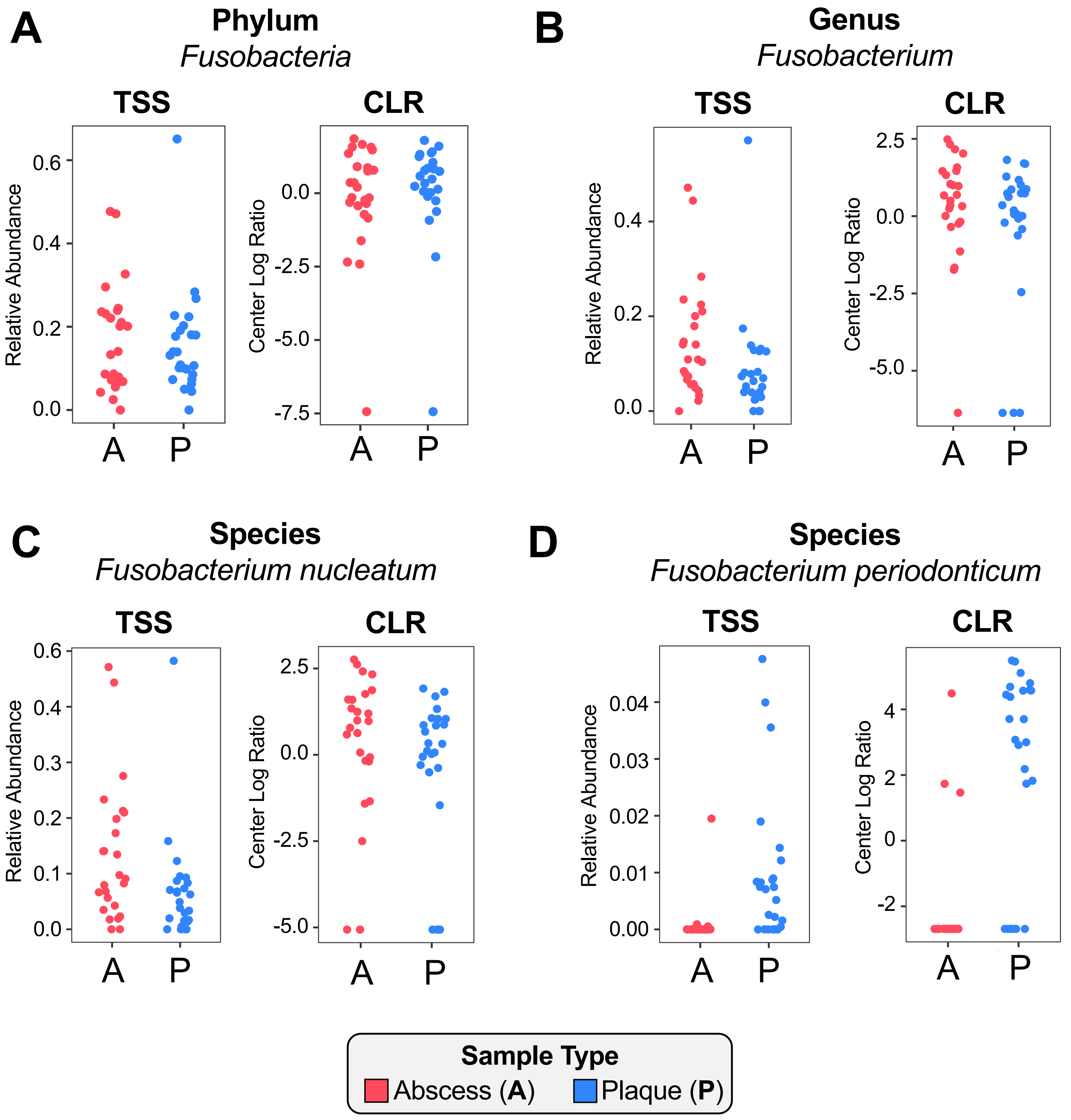
A
Phylum
Fusobacteria
TSS
CLR
Relative Abundance
Center Log Ratio
B
Genus
Fusobacterium
C
Species
Fusobacterium nucleatum
D
Species
Fusobacterium periodonticum
Sample Type
Abscess (A)
Plaque (P)

**Figure S3**

**A**

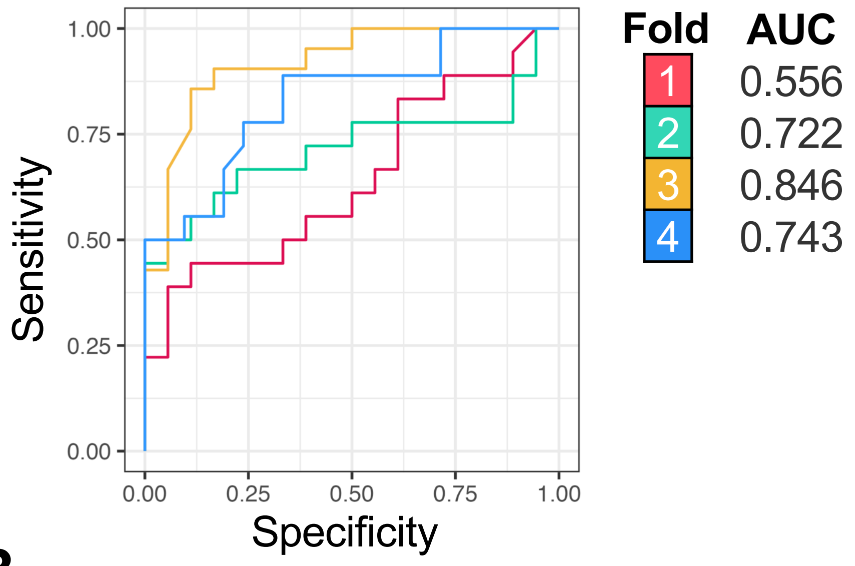


**B**

## Feature Rank per Fold

| Fold 1 | Fold 2 | Fold 3 | Fold 4 | |
|---|---|---|---|---|
| 1 | 1 | 10 | 2 | *Streptococcus sanguinis* |
| 16 | 5 | 8 | 14 | *Dialister invisus* |
| 2 | 3 | 1 | 42 | *Alloprevotella tannerae* |
| 9 | 62 | 4 | 1 | *Fusobacterium periodonticum* |
| 10 | 14 | 18 | 75 | *Neisseria elongata* |
| 24 | 60 | 36 | 6 | *Actinomyces oris* |
| 12 | 7 | 55 | 61 | *Porphyromonas endodontalis* |
| 89 | 13 | 40 | 5 | *Lautropia mirabilis* |
| 79 | 37 | 46 | 3 | *Leptotrichia sp. HMT 215* |
| 13 | 9 | 26 | 123 | *Prevotella sp. HMT 292* |
| 27 | 12 | 6 | 134 | *Rothia aeria* |
| 35 | 4 | 12 | 137 | *Prevotella oris* |
| 48 | 8 | 115 | 60 | *Neisseria flava* |
| 3 | 89 | 141 | 25 | *Veillonellaceae G-1 bacterium HMT 150* |
| 5 | 25 | 2 | 315 | *Oribacterium sp. HMT 078* |
| 33 | 22 | 297 | 7 | *Porphyromonas pasteri* |
| 319 | 32 | 3 | 9 | *Leptotrichia hofstadii* |
| 321 | 2 | 77 | 10 | *Capnocytophaga sputigena* |
| 34 | 21 | 363 | 4 | *Kingella oralis* |
| 6 | 410 | 69 | 13 | *Actinomyces massiliensis* |
| 433 | 54 | 7 | 12 | *Corynebacterium matruchotii* |
| 172 | 30 | 9 | 296 | *Lancefieldella rimae* |
| 4 | 181 | 5 | 386 | *Treponema sp. HMT 231* |
| 7 | 86 | 384 | 153 | *Abiotrophia defectiva* |
| 86 | 174 | 385 | 8 | *Arachnia propionica* |
| 8 | 390 | 80 | 303 | *Peptococcus sp. HMT 167* |
| 193 | 6 | 431 | 397 | *Neisseria oralis* |
| 346 | 10 | 440 | 291 | *Neisseria cinerea* |

**Feature Rank**
- Most important
- Least important

**Figure S4**

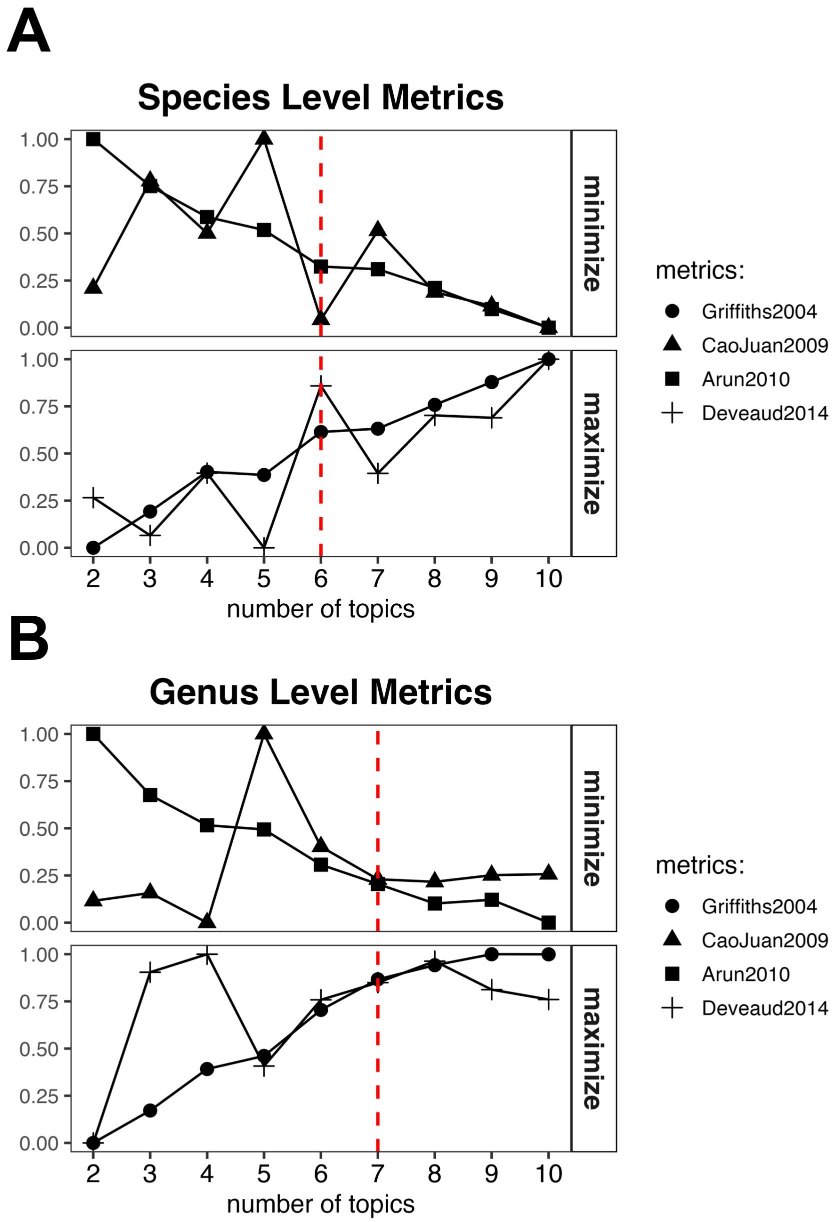
A
Species Level Metrics
1.00
0.75
0.50
0.25
0.00
minimize
maximize
2
3
4
5
6
7
8
9
10
number of topics
metrics:
Griffiths2004
CaoJuan2009
Arun2010
Deveaud2014
B
Genus Level Metrics
number of topics

# Figure S5

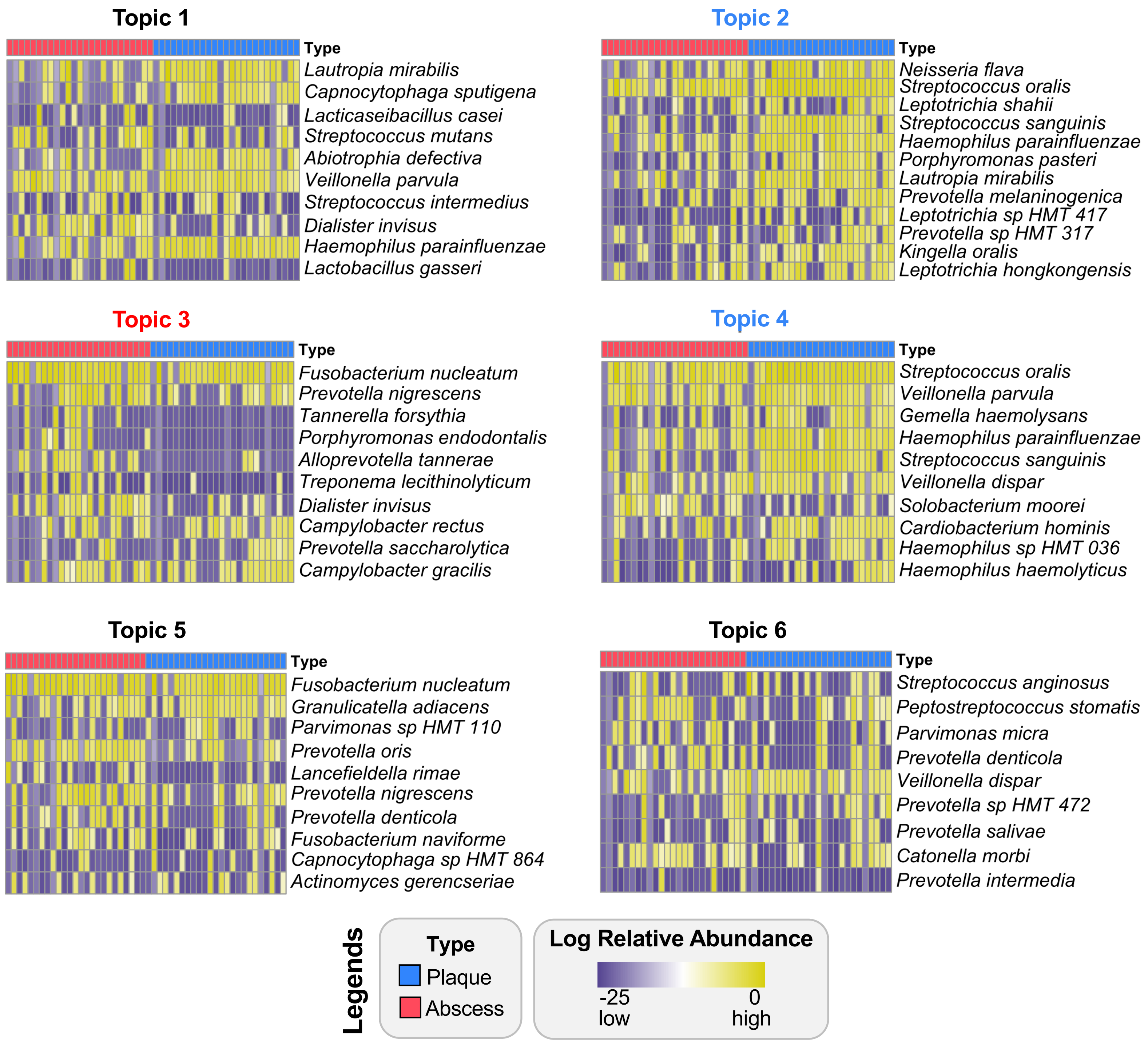

Topic 1
Type
Lautropia mirabilis
Capnocytophaga sputigena
Lacticaseibacillus casei
Streptococcus mutans
Abiotrophia defectiva
Veillonella parvula
Streptococcus intermedius
Dialister invisus
Haemophilus parainfluenzae
Lactobacillus gasseri
Topic 2
Type
Neisseria flava
Streptococcus oralis
Leptotrichia shahii
Streptococcus sanguinis
Haemophilus parainfluenzae
Porphyromonas pasteri
Lautropia mirabilis
Prevotella melaninogenica
Leptotrichia sp HMT 417
Prevotella sp HMT 317
Kingella oralis
Leptotrichia hongkongensis
Topic 3
Type
Fusobacterium nucleatum
Prevotella nigrescens
Tannerella forsythia
Porphyromonas endodontalis
Alloprevotella tannerae
Treponema lecithinolyticum
Dialister invisus
Campylobacter rectus
Prevotella saccharolytica
Campylobacter gracilis
Topic 4
Type
Streptococcus oralis
Veillonella parvula
Gemella haemolysans
Haemophilus parainfluenzae
Streptococcus sanguinis
Veillonella dispar
Solobacterium moorei
Cardiobacterium hominis
Haemophilus sp HMT 036
Haemophilus haemolyticus
Topic 5
Type
Fusobacterium nucleatum
Granulicatella adiacens
Parvimonas sp HMT 110
Prevotella oris
Lancefieldella rimae
Prevotella nigrescens
Prevotella denticola
Fusobacterium naviforme
Capnocytophaga sp HMT 864
Actinomyces gerencseriae
Topic 6
Type
Streptococcus anginosus
Peptostreptococcus stomatis
Parvimonas micra
Prevotella denticola
Veillonella dispar
Prevotella sp HMT 472
Prevotella salivae
Catonella morbi
Prevotella intermedia
Legends
Type
Plaque
Abscess
Log Relative Abundance
-25
low
0
high


**Figure S6**

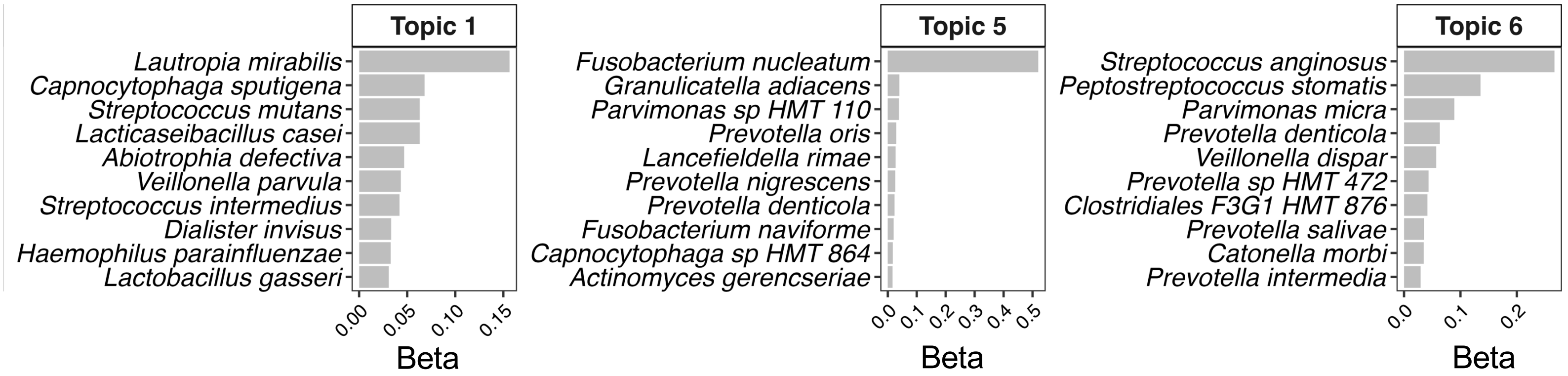

Topic 1
Lautropia mirabilis
Capnocytophaga sputigena
Streptococcus mutans
Lacticaseibacillus casei
Abiotrophia defectiva
Veillonella parvula
Streptococcus intermedius
Dialister invisus
Haemophilus parainfluenzae
Lactobacillus gasseri
0.00
0.05
0.10
0.15
Beta
Topic 5
Fusobacterium nucleatum
Granulicatella adiacens
Parvimonas sp HMT 110
Prevotella oris
Lancefieldella rimae
Prevotella nigrescens
Prevotella denticola
Fusobacterium naviforme
Capnocytophaga sp HMT 864
Actinomyces gerencseriae
0.0
0.1
0.2
0.3
0.4
0.5
Beta
Topic 6
Streptococcus anginosus
Peptostreptococcus stomatis
Parvimonas micra
Prevotella denticola
Veillonella dispar
Prevotella sp HMT 472
Clostridiales F3G1 HMT 876
Prevotella salivae
Catonella morbi
Prevotella intermedia
0.0
0.1
0.2
Beta


# Figure S7

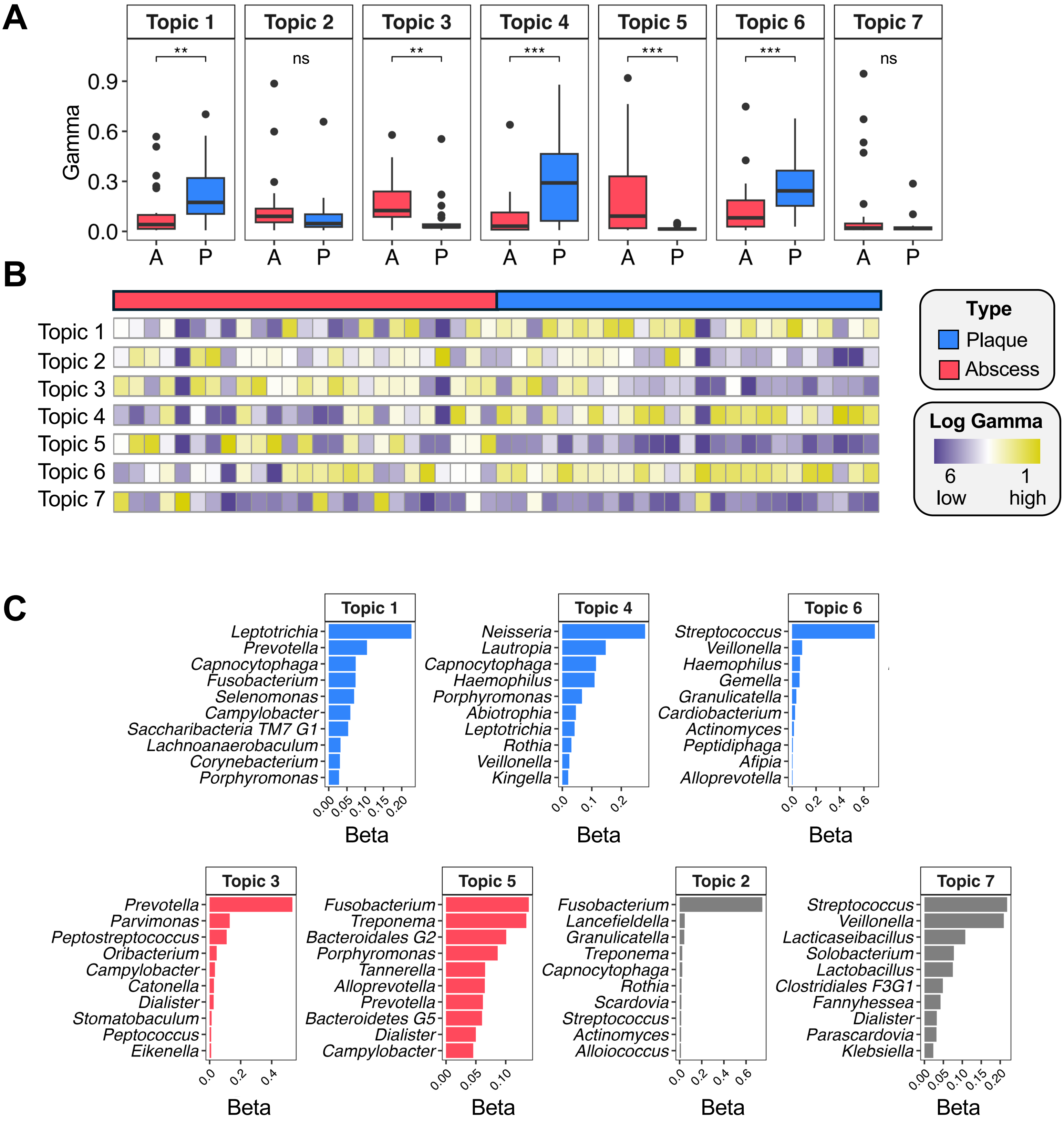

A
Topic 1
Topic 2
Topic 3
Topic 4
Topic 5
Topic 6
Topic 7
**
ns
**
***
***
***
ns
Gamma
0.9
0.6
0.3
0.0
A P
B
Topic 1
Topic 2
Topic 3
Topic 4
Topic 5
Topic 6
Topic 7
Type
Plaque
Abscess
Log Gamma
6 low
1 high
C
Topic 1
Leptotrichia
Prevotella
Capnocytophaga
Fusobacterium
Selenomonas
Campylobacter
Saccharibacteria TM7 G1
Lachnoanaerobaculum
Corynebacterium
Porphyromonas
Beta
Topic 4
Neisseria
Lautropia
Capnocytophaga
Haemophilus
Porphyromonas
Abiotrophia
Leptotrichia
Rothia
Veillonella
Kingella
Beta
Topic 6
Streptococcus
Veillonella
Haemophilus
Gemella
Granulicatella
Cardiobacterium
Actinomyces
Peptidiphaga
Afipia
Alloprevotella
Beta
Topic 3
Prevotella
Parvimonas
Peptostreptococcus
Oribacterium
Campylobacter
Catonella
Dialister
Stomatobaculum
Peptococcus
Eikenella
Beta
Topic 5
Fusobacterium
Treponema
Bacteroidales G2
Porphyromonas
Tannerella
Alloprevotella
Prevotella
Bacteroidetes G5
Dialister
Campylobacter
Beta
Topic 2
Fusobacterium
Lancefieldella
Granulicatella
Treponema
Capnocytophaga
Rothia
Scardovia
Streptococcus
Actinomyces
Alloiococcus
Beta
Topic 7
Streptococcus
Veillonella
Lacticaseibacillus
Solobacterium
Lactobacillus
Clostridiales F3G1
Fannyhessea
Dialister
Parascardovia
Klebsiella
Beta


**Figure S8**

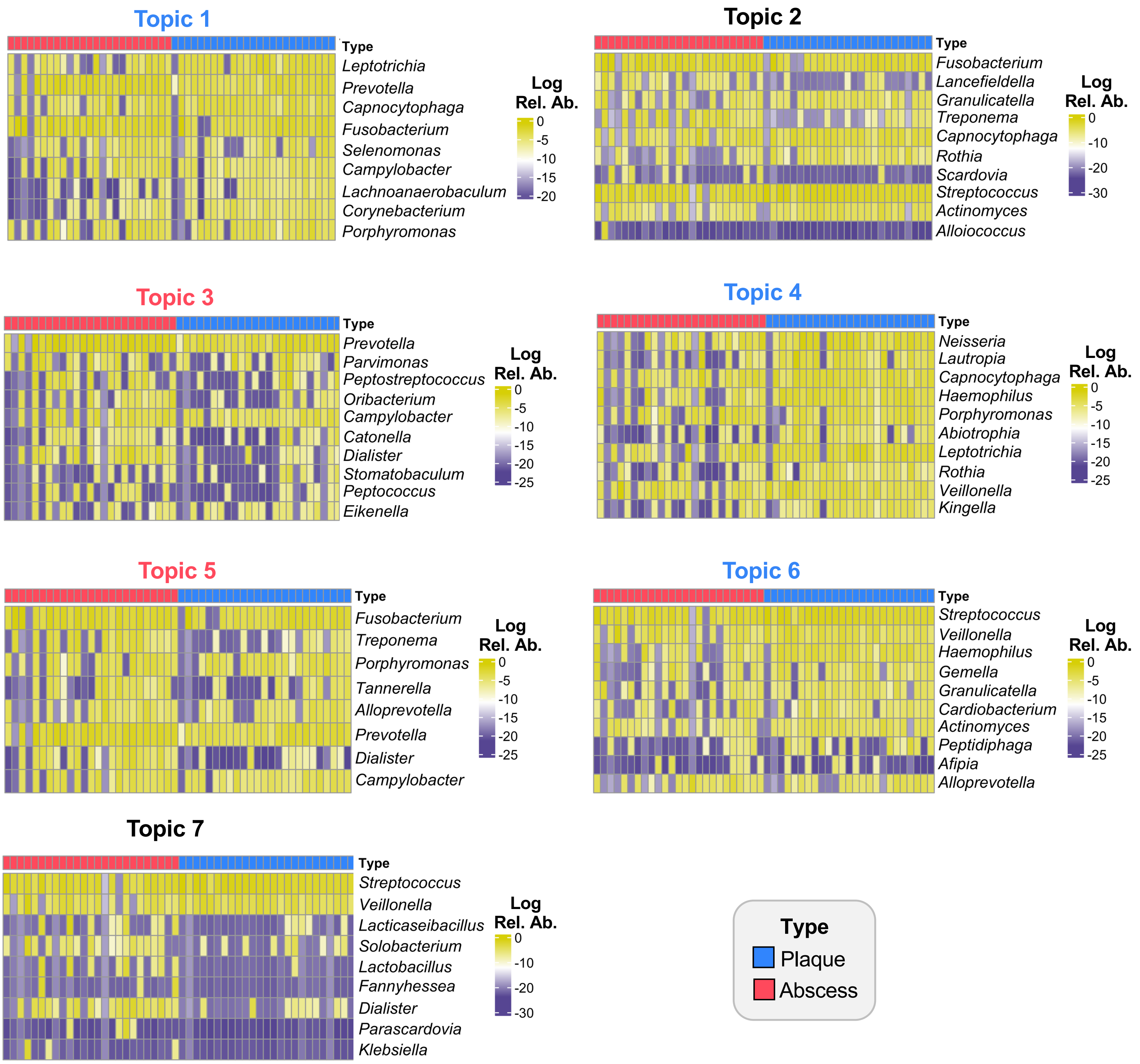
Topic 1
Type
Leptotrichia
Prevotella
Capnocytophaga
Fusobacterium
Selenomonas
Campylobacter
Lachnoanaerobaculum
Corynebacterium
Porphyromonas
Log Rel. Ab.
0
-5
-10
-15
-20
Topic 2
Type
Fusobacterium
Lancefieldella
Granulicatella
Treponema
Capnocytophaga
Rothia
Scardovia
Streptococcus
Actinomyces
Alloiococcus
Log Rel. Ab.
0
-10
-20
-30
Topic 3
Type
Prevotella
Parvimonas
Peptostreptococcus
Oribacterium
Campylobacter
Catonella
Dialister
Stomatobaculum
Peptococcus
Eikenella
Log Rel. Ab.
0
-5
-10
-15
-20
-25
Topic 4
Type
Neisseria
Lautropia
Capnocytophaga
Haemophilus
Porphyromonas
Abiotrophia
Leptotrichia
Rothia
Veillonella
Kingella
Log Rel. Ab.
0
-5
-10
-15
-20
-25
Topic 5
Type
Fusobacterium
Treponema
Porphyromonas
Tannerella
Alloprevotella
Prevotella
Dialister
Campylobacter
Log Rel. Ab.
0
-5
-10
-15
-20
-25
Topic 6
Type
Streptococcus
Veillonella
Haemophilus
Gemella
Granulicatella
Cardiobacterium
Actinomyces
Peptidiphaga
Afipia
Alloprevotella
Log Rel. Ab.
0
-5
-10
-15
-20
-25
Topic 7
Type
Streptococcus
Veillonella
Lacticaseibacillus
Solobacterium
Lactobacillus
Fannyhessea
Dialister
Parascardovia
Klebsiella
Log Rel. Ab.
0
-10
-20
-30
Type
Plaque
Abscess

**Figure S9**

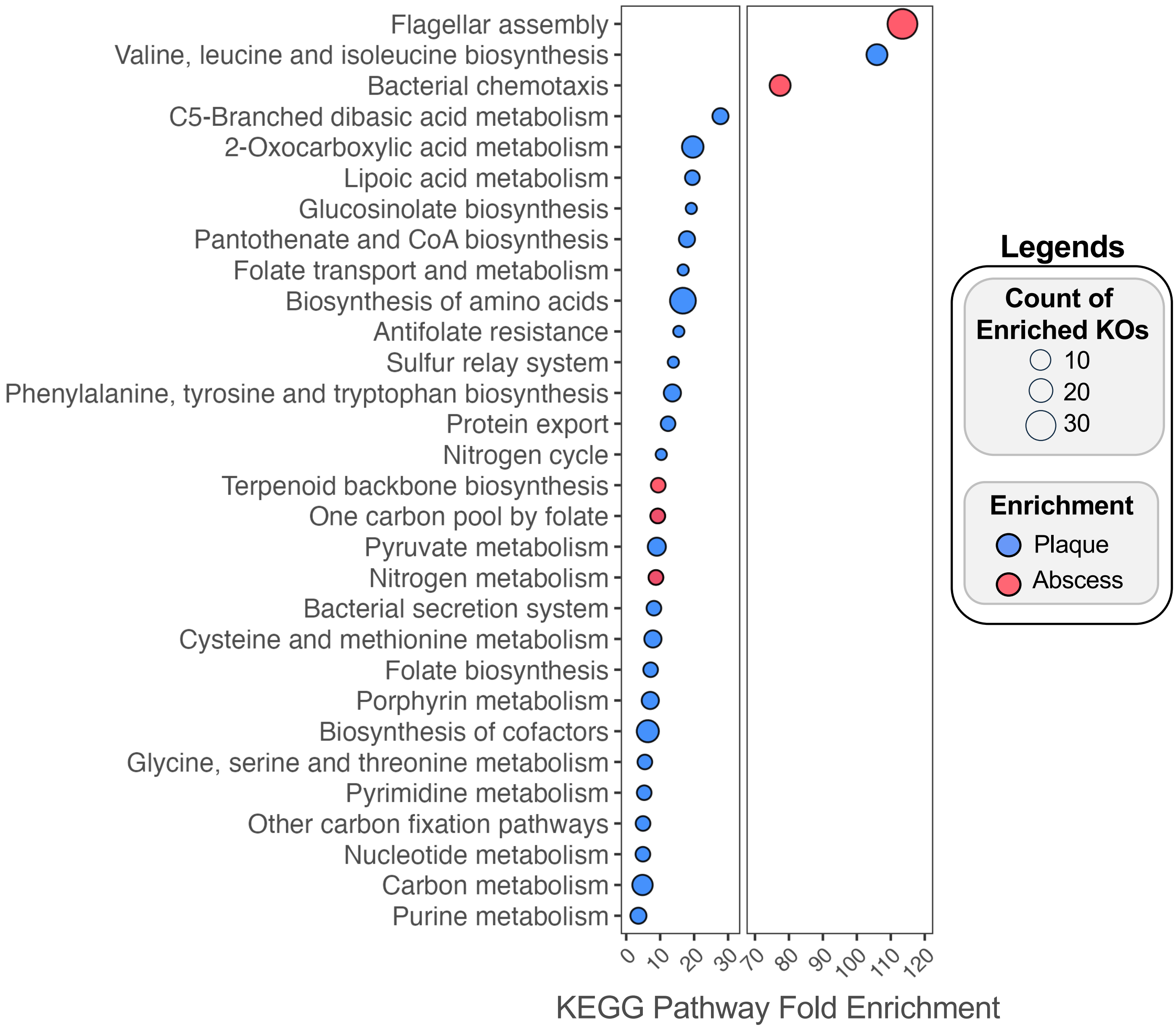
Flagellar assembly
Valine, leucine and isoleucine biosynthesis
Bacterial chemotaxis
C5-Branched dibasic acid metabolism
2-Oxocarboxylic acid metabolism
Lipoic acid metabolism
Glucosinolate biosynthesis
Pantothenate and CoA biosynthesis
Folate transport and metabolism
Biosynthesis of amino acids
Antifolate resistance
Sulfur relay system
Phenylalanine, tyrosine and tryptophan biosynthesis
Protein export
Nitrogen cycle
Terpenoid backbone biosynthesis
One carbon pool by folate
Pyruvate metabolism
Nitrogen metabolism
Bacterial secretion system
Cysteine and methionine metabolism
Folate biosynthesis
Porphyrin metabolism
Biosynthesis of cofactors
Glycine, serine and threonine metabolism
Pyrimidine metabolism
Other carbon fixation pathways
Nucleotide metabolism
Carbon metabolism
Purine metabolism
0
10
20
30
70
80
90
100
110
120
KEGG Pathway Fold Enrichment
Legends
Count of Enriched KOs
10
20
30
Enrichment
Plaque
Abscess

**Figure S10**
